# The Tailored Material Removal Distribution on Polyimide Membrane Can Be Obtained by Introducing Additional Electrodes

**DOI:** 10.3390/polym15102394

**Published:** 2023-05-21

**Authors:** Xiang Wu, Bin Fan, Qiang Xin, Guohan Gao, Peiqi Jiao, Junming Shao, Qian Luo, Zhaoyu Liang

**Affiliations:** 1 National Key Laboratory of Optical Field Manipulation Science and Technology, Chinese Academy of Sciences, Chengdu 610209, China; 2 Institute of Optics and Electronics, Chinese Academy of Sciences, Chengdu 610209, China; 3 University of Chinese Academy of Sciences, Beijing 100049, China; 4 School of Optics and Electronic Information, Huazhong University of Science and Technology, Wuhan 430074, China

**Keywords:** reactive ion etching, polyimide membrane, material removal distribution, additional electrode

## Abstract

Reactive ion etching (RIE) is a promising material removal method for processing membrane diffractive optical elements and fabrication of meter-scale aperture optical substrates because of its high-efficiency parallel processing and low surface damage. However, the non-uniformity of the etching rate in the existing RIE technology will obviously reduce the machining accuracy of diffractive elements, deteriorate the diffraction efficiency and weaken the surface convergence rate of optical substrates. In the etching process of the polyimide (PI) membrane, additional electrodes were introduced for the first time to achieve the modulation of the plasma sheath properties on the same spatial surface, thus changing the etch rate distribution. Using the additional electrode, a periodic profile structure similar to the additional electrode was successfully processed on the surface of a 200-mm diameter PI membrane substrate by a single etching iteration. By combining etching experiments with plasma discharge simulations, it is demonstrated that additional electrodes can affect the material removal distribution, and the reasons for this are analyzed and discussed. This work demonstrates the feasibility of etching rate distribution modulation based on additional electrodes, and lays a foundation for realizing tailored material removal distribution and improving etching uniformity in the future.

## 1. Introduction

Expanding the aperture of a telescope is an effective means to improve the ability of information collection and obtain higher sensitivity and resolution [1]. However, the mass of space-based optical systems increases non-linearly with the aperture. In order to reduce the cost of manufacturing as well as the limited transportation capacity of current rockets, the proposed method of using lightweight membrane diffractive lenses has brought promising prospects for reducing the mass of large-aperture optical systems [2,3,4]. Due to their low surface density, high UV/gamma radiation resistance and good mechanical and chemical stability, polyimide membranes have become an attractive candidate for making large-aperture diffractive lenses and haves been used in the construction of large space-based telescopes [5,6,7,8]. G.H. Gao studied the wet expansion behaviors of PI membranes, supported by different fixtures, and found that the Fresnel zone lens, based on a silica-fixed PI membrane, achieves near zero coefficient of wet expansion, and good imaging quality [9]. Adopting a polyimide composite membrane with SiO_2_ antireflective membrane on both sides provided a solution to improve optical homogeneity and achieve high transmittance [10]. In addition, copolyamide-imide membranes with a low coefficient of thermal expansion and coefficient of moisture expansion can be used as large aperture membrane optical system architectures [11]. However, the high-precision processing and fabrication of substrates and etching patterns for lightweight PI membrane diffractive lenses is still a challenging task. In order to improve the diffraction efficiency and imaging quality, it is necessary to figure out the original substrate of the membrane to achieve the required surface accuracy. In addition, high-precision processing of the etching pattern is extremely important.

As shown in Table 1, from the current optical processing methods, small tool polishing is usually accompanied by large contact stress, and it is not applicable to the processing of light weight films [12,13]. However, the non-contact processing methods represented by magnetorheology and ion beam have a small volume of removal function and extremely low processing efficiency for optical component substrates [14,15]. In addition, the higher plasma temperature will cause damage to the membrane substrate, and the plasma jet method is clearly not suitable for etching PI films [16]. A rapid figure correction process for PI membrane optical elements by reactive ion etching has been proposed previously [17]. However, in the full aperture range, the etching rate distribution on the membrane surface is assumed to be the same in a single processing iteration. It requires multiple applications of the mask layer. A customized material removal distribution function based on the face shape residuals has not been developed yet. This will directly enlarge the number of iterations of workpiece processing, increase the processing cost, and deteriorate the final convergence of the surface accuracy for membrane-optical elements. In addition, the high-precision machining of etching patterns needs to maintain a high uniform etching rate distribution in the chamber of etching equipment to reduce processing errors everywhere on the surface of the sample. Therefore, how to obtain the required etching rate distribution, that is, to realize the modulation of material removal distribution in the full aperture range, has become the main problem to be solved.

In recent years, the discharge characteristics and etching process of capacitively coupled reactive ion etching have been studied extensively by many scholars. The electron heating mechanism in RF capacitively coupled discharges at atmospheric to sub-atmospheric pressure was analyzed by Sanghoo Park [18]. D.W. Liu found that ohmic heating is the main mechanism of electron heating in atmospheric pressure plasma, and described the temporal and spatial evolution of electron heating in different modes [19]. E.V. Endiarova et al. improved the distribution characteristics of plasma by optimizing the geometry of the reactive ion coil [20]. Ho Jun Kim proved that the dielectric sidewall can lead to a more uniform plasma distribution than the grounded sidewall through simulation and experiment [21]. In addition, in the capacitively coupled plasma etching process, the combination of higher and lower RF frequencies, or the application of voltage phase modulation to control the ion density and ion energy respectively, are also typical methods to modulate the etching characteristics [22,23,24]. Although these methods mentioned above can enhance the uniformity of plasma distribution, there is not yet an effective, simple, and easy-to-operate means of obtaining tailored etch rate distributions in the full aperture range.

In this paper, we achieved the regulation of ion energy distribution by introducing additional electrodes to vary the sheath potential in planar space, which plays a dominant role in the etching rate, and thus achieved a tailored material removal distribution on PI membranes. The purpose of this work is to analyze the effect mechanism of additional electrodes on the plasma sheath properties on the same spatial surface by modeling the plasma discharge process for reactive ion etching, and verify the modulating effect of additional electrodes on the etching rate of membrane optical components through experiments. This provides an effective and universal solution for achieving customized material removal distribution in the full aperture range, as well as for compensating and improving the uniformity of reactive ion etching.

## 2. Experimental Setup

A homemade PI membrane (Key Laboratory of Membrane Optical Camera, Institute of Optics and Electronics Chinese Academy of Sciences, Chengdu, China) was used to study the etching behavior of RIE with additional electrodes, and the thicknesses were about 25 µm. These homemade PI membranes were synthesized by 1,2,4,5-Benzenetetracarboxylic anhydride and 2,2′-Bistrifluoromethylbenzidine, and formed homogeneous film by multiple spin coats and imidization. Young’s modulus is about 7 GPa, and the transmittance is more than 80% at 632.8 nm [9]. In order to keep the PI membrane dimensionally stable and minimize the distortion of the etched pattern, the PI membrane substrates were fixed on a special circular tooling. In order to facilitate the measurement of etch depth, a diffractive micro-structure mask pattern using a photoresist (AZ1500) was exposed on the PI membrane substrate before RIE. The photolithography process mainly includes spin coating photoresist on the PI substrate, exposure and development, hard bake, dry etching and cleaning the PI substrate [9]. During the etching process, an additional electrode made of aluminum alloy (2A12) was placed under the PI membrane substrate. After the etching process was completed, the photoresist on the PI substrate was cleaned, and the diffraction micro-structure on the PI membrane was obtained.

**Figure 1:**
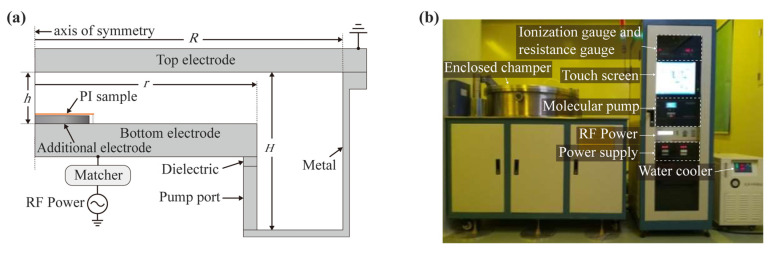
Experimental setup for RIE: (**a**) schematic diagram; (**b**) photograph.

The RIE process was performed on the apparatus shown in Figure 1a. The radius *r* of the bottom electrode is 325 mm, and it is connected to a matcher, which is connected to radio frequency (RF) power (13.56 MHz, 500 W). More information about the size of the discharge chamber, such as the electrode gap *h*, the radius *R* of the discharge chamber, etc., was provided in the literature [25]. The top electrode and the side walls are grounded. As shown in Figure 1b, RIF was performed in a customized 650-mm aperture capacitively coupled plasma (CCP) reactor (Beijing Jinsheng Weina Technology Co., Ltd, Beijing, China). During operation, the RF power generates an RF-changing electric field in the flat capacitor, which excites the gas in the chamber and generates a plasma sheath in the immediate area of the electrode. The sheath voltage drives the charged particles in the plasma toward the bottom electrode, causing them to chemically react and physically bombard the atoms on the sample surface. Finally, this volatile etching product is pumped away. In the etching process, helium was used as the discharge gas, the chamber pressure was maintained at 0.1 Torr, and the substrate temperature was controlled by a water cooler.

## 3. The Numerical Simulation of CCP with Additional Electrodes

In this paper, we use a two-dimensional fluid model to describe particle transport in CCP discharges and obtain the density, momentum, and energy of each species by solving Poisson’s equation and the first two moments of the Boltzmann equation. The electron density and average electron energy are calculated by solving their drift-diffusion equations [20,26]:

(1) $$\frac{\partial }{\partial t}\left(n_{e}\right)+\nabla \cdot \left[-n_{e}\left(\mu_{e}\times Ε\right)-D_{e}\cdot \nabla n_{e}\right]=\sum_{j=1}^{M}x_{j}k_{j}N_{n}n_{e}$$

(2) $$\frac{\partial }{\partial t}\left(n_{\varepsilon }\right)+\nabla \cdot \left[-n_{\varepsilon }\left(\mu_{\varepsilon }\cdot Ε\right)-D_{\varepsilon }\cdot \nabla n_{\varepsilon }\right]+Ε\cdot \left[-n_{e}\left(\mu_{e}\cdot Ε\right)-D_{e}\cdot \nabla n_{e}\right]=R_{\varepsilon }$$

(3) $$\bar{\varepsilon }=\frac{n_{\varepsilon }}{n_{e}}\;\mathrm{and}\;T_{e}=\frac{2}{3}\bar{\varepsilon }$$

where $n_{e}$ represents the electron density, $\mu_{e}$ the electron mobility, Ε the RF electric field, $D_{e}$ electron diffusivity, $x_{j}$ the mole fraction of the target species for reaction j, $k_{j}$ the rate coefficient for reaction j, $N_{n}$ the total neutral number density, $n_{\varepsilon }$ the electron energy density, $\mu_{\varepsilon }$ the electron energy mobility, and $T_{e}$ the electron temperature. $D_{\varepsilon }$ and $R_{\varepsilon }$ are the electron energy diffusivity and the source term for electron energy, respectively. For the self-consistent calculation of the electric field, Poisson’s equation is solved simultaneously with the fluid equation as follows [27]:

(4) $$E=-\nabla V\;\mathrm{and}\;\nabla V=-\frac{\left|q\right|}{\varepsilon_{0}}\left(n_{p}-n_{e}\right)$$

In the above equations, V is the electrostatic potential, q is the elementary charge, $\varepsilon_{0}$ is the vacuum permittivity, and $n_{p}$ is the particle density of particle p. At the driven electrode, $V=V_{rf}$. The rate constants in the model are automatically computed based on the selected electron energy distribution function (EEDF) using the formula [28]:

(5) $$k_{j}=\gamma \int_{0}^{\infty }\varepsilon \sigma_{j}\left(\varepsilon \right)f\left(\varepsilon \right)d\varepsilon \;\mathrm{and}\;\gamma ={\left(\frac{2q}{m_{e}}\right)}^{1/2}$$

Here $m_{e}$ is the electron mass, ε the electron energy, and $\sigma_{j}\left(\varepsilon \right)$ the collision cross-section, which is obtained from the Boltzmann code BOLSIG [29]. $f\left(\varepsilon \right)$ is EEDF. In this work, the chamber pressure is 100 mTorr, and the EEDF can be approximated as a Maxwell distribution function in the model [30,31]. The electron fluxes and electron energy flows that are perpendicular to the electrodes and chamber walls are as follows [26]:

(6) $$n\cdot \Gamma_{e}=\frac{1-r_{e}}{1+r_{e}}\left(\frac{1}{2}v_{e,th}n_{e}\right)-\left(\sum \gamma_{i}\left(\Gamma_{i}\cdot n\right)+\Gamma_{t}\cdot n\right)$$

(7) $$n\cdot \Gamma_{\varepsilon }=\frac{1-r_{e}}{1+r_{e}}\left(\frac{5}{6}v_{e,th}n_{\varepsilon }\right)-\left(\sum \gamma_{i}\bar{\varepsilon_{i}}\left(\Gamma_{i}\cdot n\right)+\bar{\varepsilon_{t}}\left(\Gamma_{t}\cdot n\right)\right)$$

(8) $$v_{e,th}=\sqrt{\frac{8k_{b}T_{e}}{\pi m_{e}}}$$

where $r_{e}$ represents the reflection coefficient, $v_{e,th}$ the thermal velocity, $\gamma_{i}$ the secondary emission coefficient from the *i*th positive ion species, $\Gamma_{i}$ the ion flux of the *i*th positive ion species at the wall, $\Gamma_{t}$ the thermal emission flux, $\bar{\varepsilon_{i}}$ the mean energy of the *i*th species of secondary electrons, $\bar{\varepsilon_{t}}$ the mean energy of thermally emitted electrons, n the outward normal, and $k_{b}$ is the Boltzmann constant. During the etching process, the driven electrode drives the gas discharge with a fixed power $P_{rf}$. The voltage is calculated from the following expressions and constraint sets:

(9) $$V_{rf}=V_{a}\cos\left(2\pi f_{p}t+\alpha \right)+V_{dc,b}$$

(10) $$0=f_{0}\iint_{\partial t\partial \Omega }\left(n\cdot J_{i}+n\cdot J_{e}+n\cdot J_{d}\right)dSdt$$

(11) $$P_{rf}=f_{p}\iint_{\partial t\partial \Omega }\left(V_{rf}-V_{\mathrm{dc},b}\right)\left(n\cdot J_{i}+n\cdot J_{e}+n\cdot J_{d}\right)dSdt$$

In these equations, $V_{a}$ is RF potential, $V_{dc,b}$ the DC self-bias voltage, and $J_{g}$ (*g* = *i*, *e*, *d*) is the diffusive flux of particle *g*. The various gas-phase chemical reactions considered in the reactor are shown in Table 2 [32].

## 4. Results and Discussion

**Figure 2:**
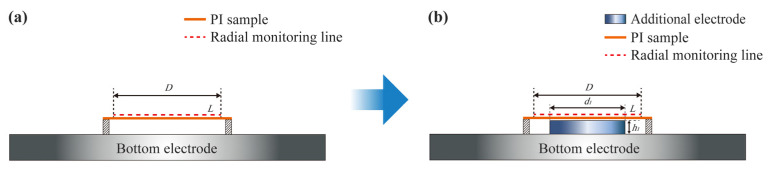
Schematic of processing PI membrane optical element without (**a**) and with (**b**) the additional electrode.

In this paper, as shown in Figure 2b, an additional electrode with a diameter of *d*_1_ = 16 mm and a thickness of *h*_1_ was placed in the center of the bottom electrode in the reactor as opposed to the general PI membrane etching process (as shown in Figure 2a). The upper surface of the additional electrode was lower than the bottom surface of the PI membrane, which was fixed on a circular tooling. In order to explore the effect of additional electrodes on the plasma sheath properties, the radial monitoring line *L*, which was 0.01 mm above the surface of the PI sample and had a diameter of *D* = 32 mm, was analyzed.

**Figure 3:**
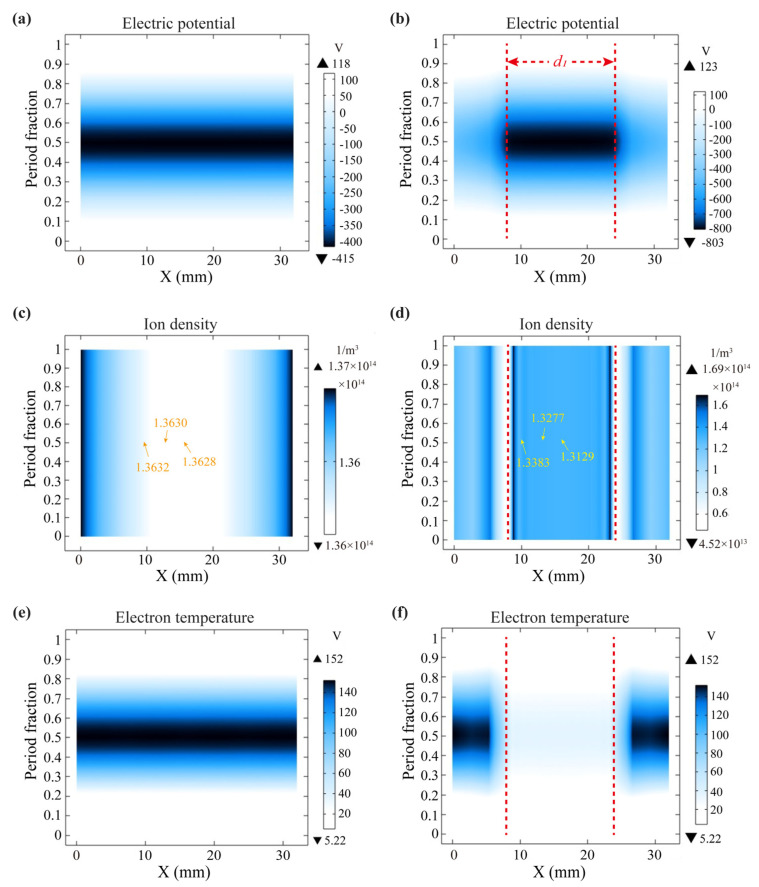
The spatio-temporal distributions of the plasma sheath potential (**a**), ion density (**c**) and electron temperature (**e**) at the radial monitoring line *L* without additional electrodes. The spatio-temporal distributions of the plasma sheath potential (**b**), ion density (**d**) and electron temperature (**f**) at the radial monitoring line *L* when using additional electrodes.

In general, reactive ion etching occurs as a result of the combined action of spontaneous chemical etching and ion-induced etching. Among them, plasma parameters such as plasma density, ion energy, and electron temperature have a great influence on the etching rate [33]. Therefore in the paper they are the main investigated plasma sheath properties. Figure 3a,c,e show the spatio-temporal distributions of the plasma sheath potential, ion density and electron temperature at the radial monitoring line *L* without the additional electrodes, respectively. These sheath properties varied periodically with RF excitation, which was consistent with the research results in the literature [19,34]. Due to the small range of the radial monitoring line *L* (*D* = 32 mm) compared to the diameter of the bottom electrode (2*r* = 650 mm), the very high uniformity of the plasma parameter distribution on the radial monitoring line *L* can be clearly observed. Figure 3b,d,f show the spatio-temporal distributions of the plasma sheath potential, ion density and electron temperature at the radial monitoring line *L* when using the additional electrodes, respectively. The width of *d*_1_ between the two red lines is the distribution of the sheath properties in the region directly above the additional electrode.

When comparing Figure 3a,b, it can be seen that the additional electrode significantly reduced the sheath potential on the same surface above the additional electrode, and the minimum potential −803 V was approximately twice the minimum sheath potential −415 V when the additional electrode was not used. At the same time, the use of additional electrodes increased the difference in sheath potential between the middle region and the side regions on the radial monitoring line *L*. In the process of RIE, the active charged particles in the plasma were driven by the sheath voltage towards the workpiece surface and undergo spontaneous chemical etching and ion-induced etching with the atoms on the workpiece surface, thus forming volatile gases, resulting in the removal of materials on the surface of the workpiece [35]. Obviously, the higher the sheath voltage, the higher the energy obtained by the active ions [36]. In Figure 3c,d, it can be seen that the use of the additional electrode resulted in a lower ion density in the region of the plasma sheath above the additional electrode, in addition to having a larger ion density variation in the edge region of the additional electrode. This was an interesting phenomenon because such a result was just the opposite of the effect of the additional electrode on the sheath voltage. In Figure 3e,f, it was found that the electron temperature above the additional electrode was significantly lower than that without the additional electrode. In addition, the extreme values of electron temperatures in Figure 3c,f are equal in an RF cycle, respectively. This may be the result of the radial monitoring line *L* being located at different spatial heights with respect to their plasma sheaths. It can be concluded from Figure 3 that the use of additional electrodes resulted in a significant change in the plasma parameter distribution, which indicated that it could effectively modulate the properties of plasma sheath in the same space plane.

**Figure 4:**
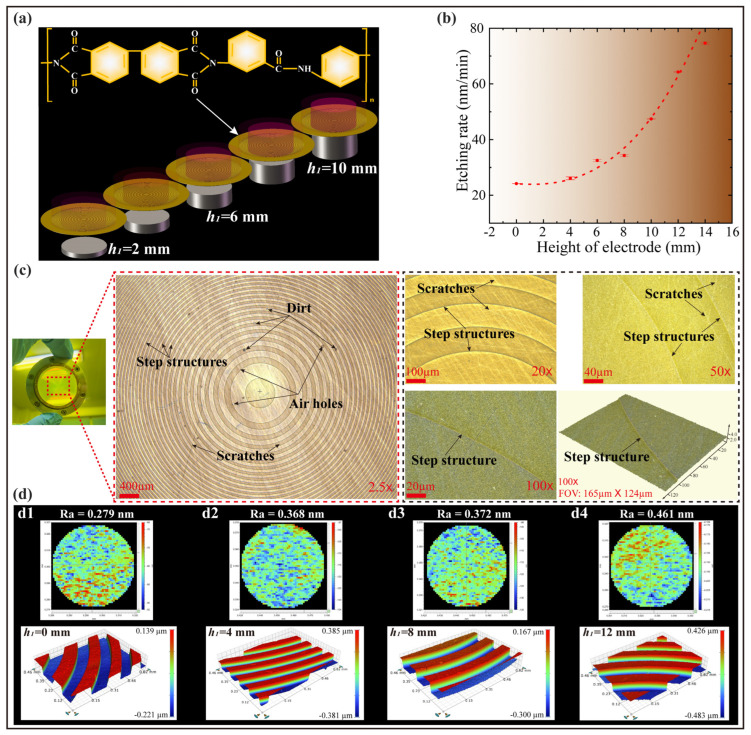
Influence of additional electrodes on etching rate: (**a**) Schematic of etching process for diffracting micro-structures on PI membrane using additional electrodes with different heights; (**b**) Influence of height of electrode on etching rate; (**c**) Photographs of PI membrane diffraction elements at different magnifications; (**d**) Surface images for samples obtained by etching using electrodes of different heights; (**d1**–**d4**) are the surface images and roughness for different electrode thicknesses *h1*, respectively.

Figure 4a shows the schematic of the etching process for diffracting micro-structures on the PI membrane using additional electrodes with different heights. The influence of the height of the electrode on the etching rate and surface profile for PI membrane optical elements is shown in Figure 4b,d respectively, and its etching depths and surface images were measured by a 3D optical surface profiler (NewView 7300 by Zygo, Middlefield, CT, USA). It can be clearly seen that the etching rate was positively correlated with the height of the additional electrode. Since the molecular formula of the homemade PI material used in the experiment was shown in Figure 4a, it is composed of C, H, N and O elements and the reaction gas was the inert gas helium, which was different from the universal Si-based material etched by fluorine-based plasma [37,38]. In the etching experiment, the active particles do not chemically react with the PI membrane and the temperature of the reactor substrate is controlled by a water cooler, so the effect of electron temperature on the etching rate was not considered here. Thus, with the use of additional electrodes, the sheath voltage dominated the etching process relative to the ion density, and the ion-induced etching influenced by the sheath voltage was able to remove the material from the PI membrane surface, i.e., the ion energy obtained through the sheath was higher than the bonding energy between the atoms of the PI membrane material. In Figure 4(d1–d4) show the surface images (0.46 mm × 0.62 mm range) and roughness (50 µm × 50 µm range) for different electrode thicknesses *h*_1_, respectively. Obviously, the surface diffraction micro-structure with a clear outline could be etched, and at the same time, it can be observed that the larger sheath voltage not only promoted the etching rate, but also increased the surface roughness to some extent. Therefore, in the future processing of PI membrane substrates, we need to find a balance between increasing etching efficiency and reducing high-frequency errors.

Figure 4c was a photograph of a PI membrane diffraction element etched using additional electrodes. These photos of the membrane surface were obtained by using an optical profiler (Zeta 3D-500 by ZETA Instrument, San Jose, CA, USA). From the morphology of the membrane surface at different magnifications, it can be seen that there was no obvious deposit except the scratches, dirt and air holes of the material itself on the surface of the PI membrane, which had obvious differences and advantages compared to the etching process using fluorine-based gas. Therefore, by using inert gas and additional electrodes to process the PI membrane, the etching rate can be flexibly adjusted, and there was almost no etching deposit attached to the surface of the sample to cause pollution.

**Figure 5:**
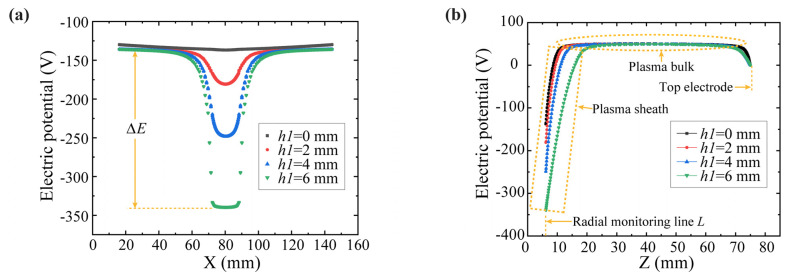
Influence of additional electrodes on sheath potential: (**a**) The time-averaged potential profiles on the radial monitoring line; (**b**) The axial time-averaged potential profiles from the top electrode to the radial monitoring line.

In order to further analyze the influence of the height of the additional electrode on the properties of the plasma sheath on the same space surface, the time-averaged potential profiles on the same radial monitoring line *L* with different heights *h*_1_ of the additional electrode are shown in Figure 5a. Here, for the convenience of observation, the width *D* of the radial monitoring line *L* was increased to 128 mm. As can be seen from Figure 5a, the sheath potential basically does not change on the two sides of the radial monitoring line *L*, while the sheath potential difference Δ*E* was generated between the central region of the radial monitoring line *L* and the regions on either side of it. This sheath potential difference Δ*E* was enhanced with the increase of the height of the additional electrodes. Figure 5b shows the influence of additional electrodes with different heights of *h*_1_ on the axial time-averaged potential profiles from the top electrode to the radial monitoring line *L* in the reactor. It can be seen that in the plasma bulk region, the plasma was almost electrically neutral [23], while the sheath potential on the radial monitoring line *L* decreased as the height of the additional electrode increased, resulting in an enhancement of the sheath voltage. These simulation results were consistent with those observed in the experiments shown in Figure 4b, i.e., the etching rate enhanced with the increase of the additional electrode height. This provided the feasibility of controlling the distribution of the etching rate by using additional electrodes.

**Figure 6:**
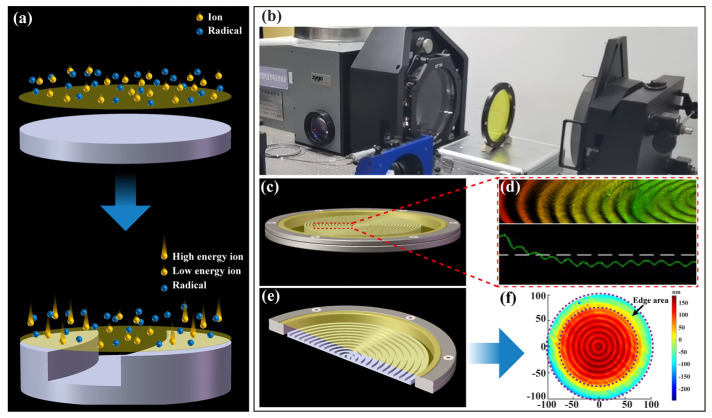
The tailored material removal distribution on PI membrane substrate: (**a**) Schematic of an effect of additional electrode on the properties of the plasma sheath; (**b**) The measurement of transmitted wave–front of PI membrane substrate; (**c**,**e**) are the additional electrodes used to etch PI membrane substrates with different periodic structural profiles on the surface, respectively; (**d**) The transmitted wave-front map of PI membrane substrate by using additional electrode (**c**); (**f**) The material removal distribution map of PI membrane substrate by using additional electrode (**e**).

Figure 6a is a schematic diagram of the influence of additional electrodes on the etching rate. Compared with the flat electrode, the spatial height difference on the surface of the additional electrode directly affects the potential distribution in the sheath region, which causes the ions in the plasma bulk region to have different ion energies when they are accelerated by the sheath voltage to the surface of the PI membrane sample, thus affecting the distribution of etching rate. In order to investigate the ability to modulate the material removal rate distribution in a large aperture range, as shown in Figure 6c,e, two additional electrodes with periodic surface profile structure with diameters of 130 mm and 187 mm were used to etch a 200-mm aperture PI membrane substrate. The transmitted wave-front maps of the PI membrane substrate before and after being etched were measured by an interferometer (GPI300 by Zygo, Middlefield, CT, USA), as shown in Figure 6b.

Figure 6d,f show the transmitted wave-front and material removal distribution of the PI membrane substrate after being etched, respectively. It is obvious from Figure 6d that a periodic contour structure similar to that of the additional electrode appears on the surface of the etched PI membrane substrate. Moreover, it can be seen from Figure 6f that the etching rate corresponding to the concave part of the additional electrode was relatively small when the edge effect of the additional electrode is not considered, while more material was removed corresponding to the convex part of the additional electrode, thus obtaining a material removal distribution influenced by the surface profile structure of the additional electrode. This indicates that the additional electrode is able to modulate the plasma sheath properties on the same spatial plane in a large aperture range, thus changing the etching rate distributions in different regions on the surface of the PI membrane sample and obtaining a tailored material removal distribution.

## 5. Conclusions

In RIE, additional electrodes with spatial height differences on the surface can change the potential distribution on the spatial plane in the plasma sheath region. In addition, in a certain range, the sheath voltage on the spatial surface increases with the enhancement of the height difference of the additional electrode surface profile, and at this time, the potential spatial difference in the sheath region dominates the etching process relative to the ion density. The use of additional electrodes to modify the sheath properties can be considered a very characteristic method to modulate the material removal distribution. In this paper, this modulation of the material removal distribution was verified by simulations and etching experiments on the PI membrane. It can be expected that the additional electrodes with different surface profiles produced by machining and 3D printing will provide a new solution for improving the uniformity of large-aperture reactive ion etching, figuring membrane-optical substrates using full-aperture removal functions, and high-precision etching of diffractive optical elements.

## Figures and Tables

**Table 1:** Material removal methods in optical substrate processing.

Material Removal Method	Processing Mechanism	Materials	Application
Small tool polishing [12,13]	Contact polishing	SiO_2_, SiC, ULE, Microcrystalline glass, etc.	Low frequency figure correction
Magnetorheological [14]	Sheared material removal	Same as above	Mid-spatial frequency figure correction
Ion beam [15]	Spot removal function	Same as above	High frequency figure correction
Plasma jet [16]	Gaussian-shaped plasma removal function	Si, SiO_2_, SiC, etc	Mid-spatial frequency figure correction
Reactive ion etching using masking [17]	Full-aperture low-temperature plasma processing	General optics, PI membrane	Low frequency figure correction
Reactive ion etching using additional electrode [This paper]	Full-aperture low-temperature plasma processing	Ultra-lightweight optics, PI membrane	Low frequency figure correction; Etching uniformity correction

**Table 2:** Plasma reactions in helium discharges.

Reaction	Formula	Type	Δε (eV)
1	e + He → e + He	Elastic	0
2	e + He → e + He*	Excitation	19.8
3	e + He → 2e + He^+^	Ionization	24.6

## Data Availability

The data are available within the article.
